# Tumor-Associated Macrophages as Multifaceted Regulators of Breast Tumor Growth

**DOI:** 10.3390/ijms22126526

**Published:** 2021-06-18

**Authors:** Maliha Tabassum Munir, Matthew K. Kay, Min H. Kang, Md Mizanur Rahman, Ahmed Al-Harrasi, Mahua Choudhury, Naima Moustaid-Moussa, Fazle Hussain, Shaikh Mizanoor Rahman

**Affiliations:** 1 Nutritional Sciences, Texas Tech University, Lubbock, TX 79409, USA; tabassum.maliha@rocketmail.com (M.T.M.); naima.moustaid-moussa@ttu.edu (N.M.-M.); 2 Obesity Research Institute, Texas Tech University, Lubbock, TX 79409, USA; 3 Texas A&M University Health Sciences Center, College Station, TX 77843, USA; matt.kay@tamu.edu (M.K.K.); mchoudhury@tamu.edu (M.C.); 4 Cancer Center, Texas Tech University Health Sciences Center, Lubbock, TX 79430, USA; min.kang@ttuhsc.edu; 5 Department of Biological and Environmental Sciences, Qatar University, Doha 2713, Qatar; mrahman@qu.edu.qa; 6 Natural and Medical Sciences Research Center, University of Nizwa, Birkat Al-Mouz 616, Oman; aharrasi@unizwa.edu.om; 7 Mechanical Engineering, Texas Tech University, Lubbock, TX 79409, USA; fazle.hussain@ttu.edu

**Keywords:** tumor-associated macrophages, breast cancer, tumor microenvironment, macrophage polarization, immunosuppression, angiogenesis, metastasis, exosomes, miRNA

## Abstract

Breast cancer is the most commonly occurring cancer in women of Western countries and is the leading cause of cancer-related mortality. The breast tumor microenvironment contains immune cells, fibroblasts, adipocytes, mesenchymal stem cells, and extracellular matrix. Among these cells, macrophages or tumor-associated macrophages (TAMs) are the major components of the breast cancer microenvironment. TAMs facilitate metastasis of the breast tumor and are responsible for poor clinical outcomes. High TAM density was also found liable for the poor prognosis of breast cancer. These observations make altering TAM function a potential therapeutic target to treat breast cancer. The present review summarizes the origin of TAMs, mechanisms of macrophage recruitment and polarization in the tumor, and the contributions of TAMs in tumor progression. We have also discussed our current knowledge about TAM-targeted therapies and the roles of miRNAs and exosomes in re-educating TAM function.

## 1. Introduction

Breast cancer is the most commonly diagnosed cancer in women around the world and the major cause of cancer-related mortality in women [1]. Although a notable decrease has been observed in the mortality rate recently, mainly because of advances in the diagnosis process and medication, there are still areas to target for treating certain types of breast cancers [2,3]. Thus, researchers are exploring potential treatment targets, such as the tumor microenvironment (TME) [2].

As early as 1889, pioneer cancer researcher Stephen Paget proposed the “seed and soil” theory and suggested that cancer cells (seed) may only induce tumor formation in the presence of a favorable microenvironment (soil) [4]. Even though cancer prevention and intervention strategies have so far emphasized cancer-cell-intrinsic factors, recent studies are focusing more on targeting the perivascular cells, endothelial cells, fibroblasts, adipocytes, and many active immune cells such as macrophages, neutrophils, and mast cells present in the TME [5,6]. Of all these cells present in the TME of breast cancer, macrophages or tumor-associated macrophages (TAMs) are the most important and account for more than 50% of the tumor mass in most human solid tumors [7,8].

Macrophages, initially described by Elie Metchnikoff studying starfish embryos in 1882, are phagocytic immune cells that provide the first line of defense, prevent infections, promote wound healing and tissue homeostasis, present foreign and self-antigens following injury, and resolve inflammation [5,9]. As macrophages show high plasticity when stimulated by various signals in the TME, they can quickly and efficiently respond to participate in innate and adaptive immune responses [2]. It is evident that the enzymes, cytokines, and chemokines (signaling proteins that can induce chemotaxis in responsive nearby cells) present in the TME would influence different functional characteristics of the macrophages present in the microenvironment [10]. Generally, macrophages are classified into two major phenotypes based on their functions—the M1 phenotype with proinflammatory responses and antitumor functions, whereas the M2 phenotype is anti-inflammatory and tumor-promoting [10,11]. Recent studies have shown that TAMs and M2 macrophages demonstrate similar functions as TAMs are of M2 phenotypes and protumoral, responding to interleukin 4 (IL-4), interleukin 10 (IL-10), transforming growth factor-beta (TGF-β), and interleukin 13 (IL-13), promoting tissue regrowth [9,12]. Further research on the interaction between cancer cells and TAMs have disclosed that TAMs not only manipulate cancer cells toward progression and metastasis but also suppress the immune responses and cause chemoresistance [2]. Thus, TAMs are viewed as an important biomarker in cancer diagnosis and a potential target for treating cancers [2]. Herein, the unique properties of TAMs in tumor progression and metastasis are discussed. Likewise, the clinical implications of TAMs as anticancer therapy are also discussed.

## 2. Macrophages in the Tumor Microenvironment (TME) or Tumor-Associated Macrophages

Tissue macrophages, which are phagocytic, play an essential role to kill pathogens and in maintaining tissue homeostasis. These macrophages can either be derived from bone marrow cells, known as recruited macrophages, or yolk sac, which is considered as resident macrophages [2,13,14].

**Figure 1:**
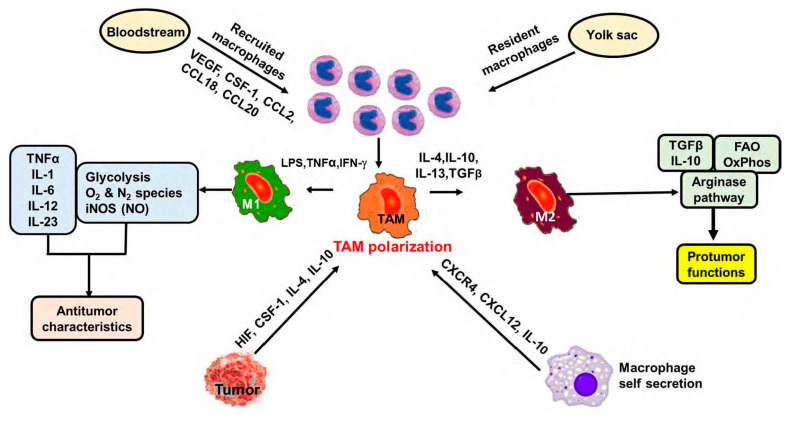
**Macrophage Recruitment and Polarization.** Monocytes from the bloodstream can be recruited to differentiate into macrophages under the influence of cytokines and growth factors such as CCL2, CCL18, CCL20, colony-stimulating factor -1 (CSF-1), and vascular endothelial growth factors (VEGFs). These recruited macrophages, along with resident macrophages, present in lung alveoli and peritoneum, epidermal Langerhans cells, Kupffer cells, and brain microglia, take part in the macrophage polarization process. Macrophages turn into either the proinflammatory M1 phenotype in the presence of LPS, TNF α, and IFN-ƴ or by, IL-4, IL-10 and IL-13 into the M2-polarized macrophages, which are generally known as TAMs. Tumors and macrophages present in the TME can release HIF, CSF-1, and interleukins CXCR4, CXCL12, and IL10, respectively, to stimulate the macrophage polarization process. Macrophages with M1 phenotype show antitumor characteristics while M2 type macrophages demonstrate protumor characteristics.

Commonly, macrophage subpopulations can be classified as either classically activated (M1) macrophages with proinflammatory yet antitumor activities; hence, they can meticulously recognize and destroy cancer cells via cytotoxicity and phagocytosis [15,16]. By contrast, alternatively activated (M2) macrophages possess anti-inflammatory features and are capable of tissue repair and growth [17]. Tumor necrosis factor alpha (TNF-α), interferon-gamma (IFN-ƴ), and lipopolysaccharide (LPS) polarize macrophages to the M1 phenotype in vitro, which regulates tumor growth and metastasis and induces Th1 response [18,19]. By contrast, interleukins (ILs) such as IL-4, IL-10, and IL-13 induce M2 macrophages [18] and are responsible for tissue remodeling, immunosuppression, and promotion of angiogenesis [18] as summarized in Figure 1.

Accumulating evidence suggests that increased M1 macrophages in the TME are associated with reduced tumor aggressiveness, while increased M2 macrophages are linked with tumor growth and poor prognosis of cancer [20]. In TME, cytokines, hormones, or apoptotic cells polarize macrophages [6,21]. Although studies showed incomplete or ambiguous results on macrophage polarization in TME, it has been established that TAMs can be both protumoral and antitumoral depending on the nature of polarization [2]. Once TAMs acquire an M2 phenotype after interacting with cancer cells, T cells or other cell types in the TME, the tumor progresses through suppression of adaptive immunity, tissue remodeling, and angiogenesis [11,22]. TAMs generally possess the M2 phenotype that is proangiogenic, although the phenotype specificity of TAMs depends on the tumor progression stage [23,24,25]. In the early stages of cancer, TAMs embrace the M1 phenotype to activate antitumor immunity and inhibit tumor angiogenesis. As the tumor progresses to advanced stages, TAMs are switched to the M2 phenotype and facilitate angiogenesis [26]. During tumor progression, M1-polarized macrophages infiltrating the tumor demonstrate a phenotype with high IL-12 and low IL-10 expressions and promote immune responses, facilitating cancer cell disruption. On the contrary, during the advanced stages of tumor progression, TAMs are of M2-like phenotype, characterized by low IL-12 and high IL-10 expressions resulting in a low tumoricidal activity [2]. These M2-like TAMs provide a microenvironment that favors tumor growth, tumor survival, and angiogenesis [5,6,10,27].

Substantial evidence supports that TAMs in breast cancer have an M2 phenotype [28]. One possible mechanism is that breast cancer cells secrete chemicals that may change those macrophages to M2 type [29]. Besides the abovementioned cytokines, tumor microenvironment signals such as hypoxia-inducible-factor-1 (HIF-1) and 2 (HIF-2), and nuclear factor–kappa beta (NF-kβ) also play an important role in TAM polarization. TAMs prefer to localize themselves to poorly vascularized cancer cells and adapt to the hypoxic environment by activating HIF-1 and HIF-2 [27,30]. Activation of hypoxia upregulates the expressions of CXC chemokine receptor 4 (CXCR4) and chemokine ligand 12 (CXCL12), which are involved in cancer metastasis [31,32,33]. Nuclear factor–kappa beta (NF-kβ) was also found to play a key role in regulating transcriptional activities of TAMs. TAMs exhibited higher IL-10 expression and reduced 1L-12 levels, which were responsible for defective NF-kβ activation [34]. Impairment in NF-kβ activation via overexpression of nuclear p50 NF-kβ homodimers inhibits the transcription of proinflammatory genes producing nitric oxide (NO), IL-1, IL-12, and TNF-α [11,34,35].

## 3. Role of TAMs in Tumor Progression

**Figure 2:**
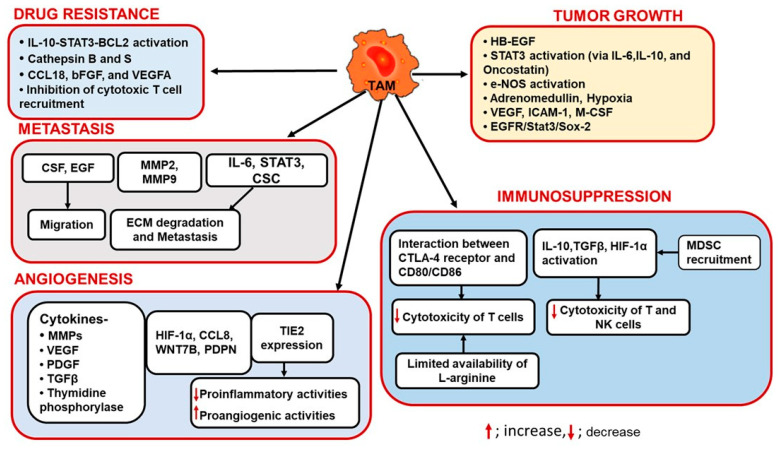
**Multifaceted Roles of TAMs in TME.** TAMS facilitate tumor growth by activating various signaling pathways including, signal transducer, and activator of transcription 3 (STAT3). The presence of M-CSF, IL-10, MMP9, and hypoxia assist TAMs in facilitating tumor growth. With tumor advancement, TAMs can either recruit immunosuppressive leukocytes or inhibit cytotoxic functions of immune cells in order to activate immunosuppression. Inhibition of cytotoxic T cell activities and recruitment causes drug resistance. Activation of the IL-10-STAT3-BCL2 pathway or cytokines and growth factors such as CCL18, basic fibroblast growth factor (bFGF), and VEGF in the TME further accelerates this TAM-mediated drug resistance. TAMs promote angiogenesis in the presence of cytokines such as MMPs and growth factors such as VEGF, PDGF, and TGFβ. TAM-mediated activation of CCL8, WNT7B, PDPN, and TIE2 expression can also lead to angiogenesis in the TME. Once angiogenesis starts, tumor and TAMs together initiate extracellular matrix (ECM) degradation resulting in tumor migration and metastasis.

TAMs which are of M2 type macrophages govern every aspect of tumor progression including angiogenesis, immunosuppression, drug resistance, and metastasis. The key findings from several studies are discussed below and summarized in Figure 2.

### 3.1. TAMs and Angiogenesis

Studies have shown that TAMs in solid tumors regulate this angiogenesis process [9]. The importance of TAMs in tumor angiogenesis was emphasized by the fact that in the presence of TAMs, the initiation of angiogenesis is facilitated [36] and depleting TAMs by the drug clodronate reduced blood vessel density in tumor tissue [37].

Numerous studies were conducted to understand the mechanisms of TAMs mediated regulation of tumor angiogenesis. Substantial evidence suggests that hypoxic regions of tumors attract TAMs and release hypoxia-induced chemoattractants such as vascular endothelial growth factor A (VEGFA), endothelins, angiopoietin2, CXCL12, and endothelial-monocyte-activating polypeptide II (EMAPII) can make TAM proangiogenic [38,39]. TAMs also secrete TNFα, matrix metalloproteinases (MMPs), VEGF, and thymidine phosphorylase, which are proangiogenic [40]. It has also been found that TAMs release growth factors such as VEGF, TGFβ, and platelet-derived growth factor (PDGF) which can facilitate angiogenesis in cancers including breast [16,36]. Moreover, it has been revealed that overexpression of CSF-1 increases the recruitment of TAMs [41], while short-interfering RNA (siRNA) mediated knock down of CSF-1 receptor decreases macrophage vascularization and infiltration in vivo [40].

TEK tyrosine kinase endothelial (TIE2) receptor is a known receptor for angiopoietins, which plays a significant role in angiogenesis [42]. One study has shown that tumor-infiltrating macrophages express TIE2, which binds with angiopoietins and facilitate angiogenesis in a mouse model of breast cancer [43].

Chemokine (C-C motif) Ligand 8 (CCL8), a chemokine produced by TAMs, was found to induce angiogenesis in breast cancer model [44]. Both in vitro and in vivo studies reveal that CCL18 and VEGF synergistically promote endothelial cell migration and angiogenesis. Additionally, the study found that blocking CCL18 or VEGF with neutralizing antibodies has a synergistic effect on inhibiting promigratory effects of TAMs. In addition, silencing phosphatidylinositol transfer membrane-associated protein 3 (PITPNM3), a chemokine (C-C motif) ligand 18 (CCL18) receptor, on the surface of human umbilical vein endothelial cells (HUVECs), can negate CCL18-mediated promigration and HUVEC tube formation. Moreover, CCL18 exposure in HUVECs caused the endothelial–mesenchymal transition and activated the extracellular signal-regulated kinase (ERK) and Akt/glycogen synthase kinase-3 beta (GSK-3β/Snail signaling, hence leading to its proangiogenic effects. Additionally, in vitro studies revealed that CCL8-mediated induction of angiogenesis in HUVEC was dependent on CCL8 receptor PITPNM3 [44]. Podoplanin (PDPN), a lymphatic endothelial cell marker, is highly expressed in TAMs and stimulates lymphangiogenesis and lymphoinvasion via activation of promigratory integrin β1 [45]. WNT family ligand WNT7B is produced in TAM and was found responsible for tumor growth and angiogenesis in a mouse breast cancer model [46]. Additionally, inhibition of WNT7B in macrophages mitigates breast tumor growth in vivo by inhibition angiogenic switch and reducing VEGFA [46].

All together, these studies underscore the critical role of TAMs in breast tumor angiogenesis. However, more studies are required to understand whether TAM-secreted molecules that dictate angiogenesis vary during breast tumor progression and the molecular details of their regulations.

### 3.2. TAMs and Immunosuppression

M1-type macrophages in the tumor display tumoricidal function via intimate interactions between innate and adaptive immunity and by inducing lysis, apoptosis, and phagocytosis of malignant cells [5]. However, macrophages infiltrating mammary tumors can also depict immunosuppressive characteristics [5]. This immunosuppression occurs through several mechanisms:

In TME, TAMs facilitate the immune shift of the tumor cells by releasing anti-inflammatory cytokines such as TGF-β and IL-10, which subscribe to the suppression of effector T cell and natural killer (NK) cell cytotoxicity [47]. In vivo studies using a mouse model of breast cancer showed TAMs weaken the cluster of differentiation 8 (CD8)+ T-cell activation and proliferation through IL-10, yet removal of TAMs from mammary adenocarcinomas with colony-stimulating factor 1 receptor (CSF1R) signaling agonist not only increased antitumor CD8+ T-cell immunity but also improved chemosensitivity [48,49]. One study has shown that IL-10 produced by TAMs in a mouse model of breast cancer represses CD8+ T-cell response concurrent with the inhibition IL-12 from dendritic cells [48].

In addition, macrophages from hypoxic tumor areas activate HIF-1α, which mediates the expression of inhibitory receptors of T-cell regulation and thus sponsors the dysfunction of tumor-specific T cells [50]. Furthermore, interaction between inhibitory cytotoxic T-lymphocyte-associated protein 4 (CTLA-4) receptor on the active T cell surfaces, and TAMs-expressing cluster of differentiation 80 (CD80) and cluster of differentiation 86 (CD86) showed reductions in cytotoxicity, and inhibition of T cell activation and cell-cycle arrest [5].

More importantly, the metabolism of L-arginine is predominantly responsible for TAM-mediated T-cell inhibition [5]. TAMs produce arginase 1 in response to IL-4, IL-10, IL-13, HIF-1α, and lactic acid, causing the catabolism of L-arginine and limiting its availability for T-cell function [51]. Moreover, L-arginine functions as the substrate for the inducible iNOS enzyme, responsible for the cytotoxic role of macrophages [5]. TAM was found to suppress MHC1 class II in murine and human breast cancer cells, which may impair antigen presentation and T cell activation [52].

TAMs also promote immunosuppression of the cancer cells through recruiting immunosuppressive leukocytes to the TME. During chronic inflammation of the TME, anti-inflammatory and tissue-repairing cells are infiltrated to the tumor stroma [5]. At the same time, the production of chemoattractants can also help TAMs to recruit immunosuppressive cells such as myeloid-derived suppressor cells (MDSCs) comprising of granulocytes, monocytes, dendritic cells, and Tregs to tumors to inhibit a cytotoxic T-cell response [5]. TAM may interact with neutrophils and can promote immunosuppression in cancer. Myeloperoxidase (MPO) is an enzyme secreted by activated neutrophils during inflammation [53]. Importantly, MPO-positive cell infiltration to colorectal cancer was associated with a favorable prognosis [54]. A study also revealed the increased presence of MPO-positive neutrophils in breast tumor tissues [55].

All of the evidence suggests that TAMs secrete molecules that make the macrophages less tumoricidal in TME and, hence, result in immunosuppression and tumor growth.

### 3.3. TAMs in Tumor Growth

Both STAT3 and EGFR play critical roles in cancer progression. Given the important role of tumor-cells–macrophages interaction on tumor growth, Phillip and colleagues investigated the STAT3 activators and EGFR agonists secreted by tumor-primed monocytes and MΦ and their impact on breast tumor growth [56]. They reported that monocytes secrete epiregulin (EREG) and oncostatin-M (OSM), a STAT3 activator of the IL-6 family, while macrophages were found to release heparin-binding EGF-like growth factor (HB-EGF), and OSM upon priming with MDA-MB-231 supernatants. They also found that TAM-derived OSM and HB-EGF promote breast cancer cell migration in vitro. Additionally, HB-EGF and OSM were coexpressed by TAMs in breast carcinoma patients. Importantly, increased level of HB-EGF was associated with TAM infiltration and tumor growth [56,57].

Studies have revealed that adrenomedullin derived from TAMs promotes tumor growth through activation of the endothelial nitric oxide synthase (eNOS) signaling pathway, and inhibition of adrenomedullin receptors on TAMs ultimately suppresses tumor growth [58,59].

Hypoxia or lower oxygen concentration in tumor regions has been found as a critical regulator of tumor progression [13]. In breast cancer, the hypoxic environment influences macrophage recruitment to the tumor site [38]. The entrapment of macrophages can be explained by the dephosphorylation of chemoattractant receptors for VEGF and C-C motif chemokine ligand 2 (CCL2), vascular endothelial growth factor receptor (VEGFR), and C-C motif chemokine receptor 2 (CCR2), respectively, and abortion of their chemotactic response in TAMs [60,61].

TAMs can regulate tumor growth by manipulating the populations of cancer stem cells (CSCs). Studies have shown that interaction between macrophages and tumor cells through macrophage colony-stimulating factor (M-CSF), intracellular adhesion molecule-1 (ICAM-1), and ephrin can increase the survival, renewal, and tumorigenic characteristics of CSCs, which consequently lead to tumor growth and chemoresistance [42,62]. TAM was also found to promote CSC phenotype and tumorigenesis in breast cancer by activating the EGFR/Stat3/Sox-2 signaling pathway, while inhibition of Sox2 mitigates TAM mediated induction of CSC phenotype and breast tumor growth [63]. A study has shown that TAM interacts with breast cancer CSC via EphA4 and enhanced cytokine signaling to maintain the CSC phenotype and tumor growth [62].

### 3.4. TAMs and Drug Resistance

Usually, a macrophage population enriched with the M2 phenotype can lead to therapeutic resistance [2]. Anticancer action of the drug Docetaxel lies within the activation of M1 macrophages and depletion of M2 macrophages, associating the role of TAMs in the therapeutic response of breast cancer [64]. In node-positive breast cancer, patients who had high counts of CD8+ T cells, but low macrophages had more recurrence-free survival than patients who had high macrophages but low CD8+ T cells and had undergone intense chemotherapy [65]. Worse yet, TAMs have also been found to show resistance against the drug tamoxifen in postmenopausal women with breast cancer [66].

Yang et al. reported that TAMs secrete IL-10 which upregulates B-cell lymphoma 2 (BCL-2) and STAT3 expressions and as a result activates the IL-10-STAT3-BCL2 pathway in breast cancer cells, which increases drug resistance [67]. TAMs were also found to be associated with tamoxifen resistance in postmenopausal breast cancer patients. The same study observed higher expression of epidermal growth factor receptor (EGFR) and CD163+ macrophages via immunostaining in a tamoxifen-resistant group compared to a tamoxifen-sensitive group [66].

TAMs secrete chemoprotective factors such as cathepsins B and S which directly protect the tumor cells from the cytotoxic effects of chemotherapeutic agents [68]. TAMs can also inhibit the recruitment of the CD8+ cytotoxic T-cells and thus induce drug resistance [65]. Interestingly, TAMs in breast cancer exhibit resistance against antiangiogenic therapies by releasing CCL18, VEGFA, and bFGF and promoting angiogenesis [20]. The angiogenesis-associated abnormal vascularization leads to suppression and resistance of chemotherapy [2]. Downregulation of these proangiogenic factors can reduce tumor vessel density and eventually increase the efficiency of therapeutic delivery to the tumors [69].

### 3.5. TAMs and Tumor Metastasis

Gorelik et al. showed the contribution of TAMs in promoting tumor metastasis [70]. Since then, a number of studies were conducted to unravel the underlying molecular mechanisms. Wyckoff et al. revealed the presence of a symbiotic relationship between cancer cells and TAMs in cancer cell migration [71]. This study further reported that both tumor cells and TAMs release CSF-1 and epidermal growth factor (EGF), respectively, which may interact which each other and facilitate the tumor cell migration [72]. Accumulating evidence suggests that the interaction between cancer cells and macrophages that remain in close proximity of TAMs is critical for the intravasation of cancer cells [73]. To determine if intravasation efficiency is associated with the density of perivascular macrophages, Csf1op/Csf1op/PyMT mice defective in CSF-1 production were used. These mice showed tumor growth like wild-type (*+/op/PyMT)* animals but had slower tumor progression and decreased invasion and metastasis consistent with the requirement for CSF-1 signaling for invasion and metastasis. Interestingly, low-density tumor-associated macrophages were found in Csf1op/Csf1op/PyMT mammary tumors. On the other hand, inhibition of CSF-1 signals lowered the number of circulating cancer cells and tumor metastasis in vivo [73].

TAMs also regulate tumor metastasis by influencing the TME. Hagemann et al. showed that the coculture of tumor cells and macrophages together increases the expression of MMP2 and MMP9, which eventually degrade the proteins present in the extracellular matrix and promote metastasis [74,75].

In addition, macrophages are recruited in the metastatic site of breast cancer and are known as metastasis-associated macrophages (MAMs). These MAMs promote extravasation and persistent growth of breast cancer cells via activating chemokine signaling. A study has shown that metastasis-associated macrophages stimulate CCL3 via secretion of CCL2 receptor CCR2 and promote lung metastasis in breast cancer [76]. Additionally, deletion of the CCL3 receptor CCR1 prevented lung metastasis and suppressed the accumulation of metastasis-associated macrophages in a mouse breast cancer model. Moreover, CCR1 inhibition suppressed the interaction between cancer cells and metastasis associated macrophages [76]. The results suggest that activation of CCL2/CCR2 provokes a signaling cascade that promotes metastasis via retention of metastasis-associated macrophages. Chen et al. reported that VCAM1 secreted by breast cancer cells connects MAMs with cancer cells via α4 integrins and promotes survival of breast cancer cells in lungs by activating Ezrin and PI3K/Akt signaling [77].

TAMs play a prominent role to facilitate metastasis via interaction with CSCs. Zhou et al. evaluated the interaction between TAMs and CSCs in breast cancer recurrence and metastasis. To mimic the breast cancer microenvironment treated with chemotherapy, MCF-7 was cultured with conditioned media from macrophages cocultured with apoptotic MCF-7 cells. They found an increased proportion of cancer stem cells accompanied with tumor growth and metastasis in a mouse model of breast cancer [78]. Their group further observed that exposure of macrophages to apoptotic MCF-7 cells increases the production of IL-6 and activation of STAT3, which may be responsible for increased cancer stem cell populations and metastasis. The results and evidence presented above clearly support a protumoral function of TAMs. Thus, targeting TAMs may be an attractive therapeutic option to prevent and treat breast cancer.

## 4. Targeting TAMs—A Useful Anticancer Therapy

**Figure 3:**
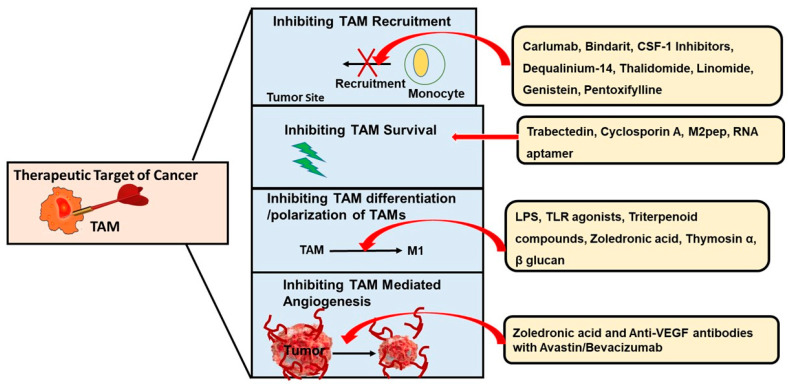
**TAMs Targeted Anticancer Therapy.** The variable role of TAMs in the TME makes them a potential target to fight against cancer progression. The first approach can be to inhibit TAM recruitment to the tumor site. Drugs such as Carlumab, Bindarit, Thalidomide, etc., can inhibit monocyte recruitment to the tumor site so that they cannot convert into TAMs and cause tumor growth. Trabectedin and Cyclosporin A inhibit tumor growth by targeting or killing TAMs or downregulating TAM survival through different mechanisms. Another approach to prevent TAM-mediated tumor progression is to reduce its differentiation and polarization. LPS and TLR agonists can reverse macrophage polarization by converting them back to M1 antitumor phenotype, while Triterpenoid compounds have been found to inhibit the initial polarization of M1 macrophages to M2 phenotype. Lastly, inhibition of angiogenesis can be a potential approach to treat tumor progression. The use of anti-VEGF-antibody in combination with Avastin, Bevacizumab, or other neutralizing antibodies can inhibit macrophage infiltration as well as angiogenesis.

A growing body of evidence now supports that TAM activation in cancer can lead to poor disease prognosis and chemoresistance [65,66] through their tumor-promoting [42,62] and immunosuppressive actions [5,47]. Because of this reason, TAMs have become a potential target for therapeutic intervention to treat different forms of cancers, including breast cancer. However, to make these therapeutic strategies efficient, it is important to understand the interaction between tumor and macrophage [2]. Recent studies have successfully shown several possible approaches in experimental settings as described below and summarized in Figure 3. Some of these approaches are now awaiting clinical trials.

### 4.1. Inhibiting the Recruitment of TAMs

Since macrophage infiltration is associated with higher progression of cancer, the first and fundamental approach to reduce the levels of TAMs would be to inhibit their recruitment to the tumor site [2]. The recruitment of circulating monocytes or macrophages to the tumor tissue is modulated by macrophage chemoattractant molecules such as CCL2 in the TME [79]. Studies have shown that in breast cancer models, inhibition of CCL2 with anti-CCL2 antibodies decreased both tumor growth and spread [76]. Even when the monoclonal therapy against CCL2 (Carlumab) was combined with other chemotherapies in patients with solid tumors, the treatment regimen was well tolerated [80]. In the case of ER-positive breast cancers, estradiol increased macrophage influx and angiogenesis in vivo by enhancing release of CCL2, CCL5, and epidermal growth factor (EGF). Inhibition of CCL2 and CCL5 showed anticancer effects by reducing macrophage infiltration and angiogenesis [81]. In triple-negative breast cancer (TNBC), anticathepsin D antibody has been found to inhibit TAM recruitment by lowering TGFß levels and hence inhibit tumor growth [82].

Recently, one study showed that treating breast cancer with Paclitaxel can increase macrophage chemotactic factors such as CCL8, IL-34, CSF-1, and CSF-1 receptors in vivo [49] to enhance TAM migration. Administration of CSF-1 inhibitors along with chemotherapy not only increased the T cell numbers in the tumors and therapeutic efficiency of the treatment but also inhibited metastasis [49].

### 4.2. Blocking the Survival of TAMs

Synthetic or chemical drugs that can cause apoptosis might be helpful for lowering the survival of TAMs [83]. In an in vivo study, Roth et al. inhibited the IL-4 receptor α (IL4Rα) using a ribonucleic acid (RNA) aptamer on ILRα^-^/^-^Balb/C4T1 mice, with a view toward targeting and eradicating TAMs [84]. This treatment increased the number of T cells, eliminated TAMs, and reduced tumor growth in tumor-bearing mice by targeting the ILRα-STAT6 signaling pathway [84]. Moreover, M2pep, a unique peptide containing a proapoptotic peptide in its structure, has been found to target and kill TAMs in a selective manner, and as a result increased the survival rate of the tumor-bearing mice. The study explained that this peptide preferentially binds to murine TAMs in vivo and has low affinity for other leukocytes. TAM-targeted delivery of the proapoptotic peptide alone, without an anticancer agent, was found sufficient to delay mortality and selectively reduced the M2-like TAM population. [85].

Another anticancer agent, Trabectedin, showed its TAM depleting efficiency by inducing apoptosis (caspase-8 dependent) via TNF-related apoptosis-inducing ligand (TRAIL) receptors. Unfortunately, this agent does not work selectively, meaning it not only affects TAMs but also restricts macrophage-mediated host defense [86]. Molecules such as M2pep, Trabectedin, and Cyclosporin A directly impede tumor growth, inhibit macrophage differentiation to the M2 phenotype, and also suppress TAM activation [20].

### 4.3. Preventing the Differentiation and Polarization of TAMs

As mentioned earlier, two major types of macrophages, M1, and M2 demonstrate very different functions. M1 macrophages show antitumor, while M2 macrophages exhibit protumor and immunosuppressive functions [10]. On that account, various studies have used different agents to reprogram the macrophages from M2 phenotype to M1 phenotype.

Liposomal Zoledronic acid depleted TAMS, reduced M2 marker CD206, inhibited CD31 expression, and ultimately reduced angiogenesis and breast tumor growth in triple-negative breast cancer [87]. Like Zoledronic acid, liposomal nanoparticle-delivered guanosine monophosphate–adenosine monophosphate (GAMP) also showed potential in TNBC growth suppression by reprogramming M2 macrophages back to M1 phenotype [88]. C-terminal fragment of adhesion protein Fibulin7 (Fbln7-C) attenuated (MDA-MB-231) supernatant-induced reeducating of human monocytes into tumor-promoting TAMs by increasing pERK1/2 and pSTAT1 expression and reducing CD206 protein expression [89]. Interestingly, Fibulin7 also reduced tumor growth by increasing inflammatory monocytes (F480^+^ Ly6C^hi^ CD11b^+^) and decreasing TAMs and anti-inflammatory macrophage marker CD206 [89].

Regulation of the signal transduction pathways might affect macrophage polarization [20]. NF-kβ, STAT3, and SAT6 can regulate TAM differentiation into M2 phenotype [2]. Thus, inhibition of these pathways may have therapeutic potentials to prevent tumor progression. Other immunomodulating agents, such as thymosin-α and B-glucan, have successfully demonstrated the reverse polarization of macrophages into M1 phenotype in vivo [90]. An herb couplet of *Hedyotis diffusa* and *Scutellaria barbata* (YDW11) was found to inhibit TAM polarization toward M2 phenotype in vitro, resulting in inhibition of breast cancer cell migration [91]. Michael and colleagues investigated the potential of synthetic oleanane triterpenoid CDDO-methyl ester (CDDO-Me) in regulating TAM function in vivo. PyMT+/− female mice fed a CDDO-Me diet demonstrated suppression of TAM infiltration to mammary tumor concomitant with reduced expression of macrophage chemoattractant CCL2 in TAMs compared to the control diet fed mice [92]. Additionally, the CDDO-Me diet significantly reduced markers of alternatively activated macrophages including IL-10 and Arg1 expression, while increasing TNFα expression and attenuating immunosuppression. More interestingly, CDDO-Me-diet-fed mice demonstrated higher CXCL16 expression and increased recruitment of activated T cells [92]. The results indicate that CDDO-Me is effective to attenuate TAM-mediated immunosuppression by increasing activated T cells in tumors.

Macrophage inhibitory factor (MIF) was also found to be effective in regulating macrophage polarization. MIF locates in the solid tumors and shifts macrophages to M2 type by modulating macrophage function [18]. siRNA-mediated knockdown of MIF in TAMs significantly reduced CD74 and CD206 while increasing TNFα and IL-2 [52,93]. Interestingly, siRNA-MIF-loaded nanoparticles injection into 4T1 tumor in mice significantly reduced tumor growth concurrent with increased infiltration of CD4 T-cells to tumor and reduction of myeloid-derived suppressive cells in the circulation [52,93].

It has also been shown that a DNA vaccine against the cysteine protease legumain, which is overexpressed in TAMs, showed potential in decreasing TAM density in the tumor tissues and inhibited tumor growth, angiogenesis, and metastasis [94].

Lastly, targeting the CSF-1/colony-stimulating factor-1 receptor (CSF-1R) signaling might be an effective approach to govern macrophage function. This pathway is associated with the alteration of macrophage polarization and macrophage survival. Activation of this pathway or a higher expression of either CSF-1 or CSF-1R results in poor prognosis of breast cancer in postmenopausal women [2,95]. Interestingly, deletion of CSF-1 not only reduced the incidence of breast cancer and delayed tumor progression but also decreased metastasis [79,96]. In clinical trials, treatment with monoclonal antibody (RG7155) blocked dimerization and activation of CSF-1 receptor, decreased TAM infiltration, and increased T cell numbers in patients [96].

### 4.4. Inhibiting TAM-Mediated Angiogenesis

TAMs facilitate the angiogenesis process. Thus, inhibition of the angiogenesis by regulating TAMs function can be a potential therapeutic approach to treat tumor progression. A few studies found that use of anti-VEGF-antibody in combination with Avastin or Bevacizumab can inhibit macrophage infiltration and at the same time can prevent TAMs from releasing proangiogenic factors, which in turn enhance the effectiveness of antiangiogenic therapies [97,98]. Use of bisphosphonates such as Zoledronic acid (ZA) was also found to decrease TAM infiltration into the tumor site and impair angiogenesis by inducing apoptosis [18]. Another study showed that ZA targets the local microenvironment and inhibits spontaneous mammary carcinogenesis by decreasing tumor vascularization, reducing the number of tumor-associated macrophages and their reverted polarization from M2 to M1 phenotype [99].

Certain products released by TAMs in the TME can also inhibit TAM-mediated tumor growth. CXCL1 inhibitor, XIAOPI formula, released from TAMs can inhibit premetastatic niche formation [100], cancel cell proliferation and thereby cancer metastasis in breast cancer [101]. A specific inhibitor of heat-shock protein 32 (HSP32) and heme oxygenase-1 (HO-1), zinc protoporphyrin IX (ZnPPIX) has also demonstrated potential against breast cancer growth. Deng et al. used ZnPPIX to evaluate its potential effects on mouse breast cancer and tumor-associated macrophages (TAMs). Their results showed that mouse 4T1 breast cancer growth can be suppressed through inhibition of HO-1 both in vitro and in vivo. Moreover, when HO-1 was suppressed in TAMs in the 4T1 mouse model, M2 type macrophages switched to M1 type. Additionally, inhibition of HO-1 might have induced tumor-associated immune response by activating TAMs’ alternative proliferation, suggesting HO-1 as an important target of breast cancer treatment [102].

## 5. miRNAs Regulate TAM Functions and Breast Cancer Pathogenesis

MicroRNAs (miRNAs) are small endogenous 19–24 nucleotide long non-coding RNAs that take part in post-transcriptional regulation by targeting messenger RNA sequences [103]. Mature miRNAs bind to the 3′ UTR of target mRNAs to degrade it or inhibit its post-transcription processing. The regulatory network between specific genes and miRNAs is versatile because multiple miRNAs can modulate a single gene, while a single miRNA can target multiple genes [104]. They regulate physiological and pathological processes such as cell division, tumorigenesis, metastasis, and the inflammatory response [105]. As such, miRNAs can play a role in controlling TAM functions and cancer progression. As part of the regulatory abilities of miRNAs, the dysregulation of miRNAs has been shown to play a significant role in cancer, including breast cancer [106,107,108]. However, its role in the regulation of TAMs and its overall impact on breast tumor growth are an emerging area of research.

miRNAs also enact a critical aspect in monocyte differentiation and macrophage polarization. Recently, the position of miRNAs in modulating macrophage behavior in tumor environments and their significance on tumor progression have generated curiosity among scientists [109,110,111]. As plasticity is an important characteristic of macrophages, TAMs can be activated in classical (M1) or alternative (M2) pathways, providing divergent regulatory functions in the TME through contrasting intracellular signaling pathways [112]. Continual recruitment of precursor cells to the TME is also essential to restock the macrophage populations [113], which is also controlled through specific miRNAs [114,115,116]. These miRNA expression profiles have also been identified through comparative studies with M1 vs. M2 macrophages [117,118,119]. Wang et al. demonstrated higher expression of miR-100 in TAMs of mouse and human breast cancer [120]. Their group also discovered that overexpression of miR-100 promoted the TAMs phenotype via regulation of the mTOR pathway. They further reported attenuation of TAMs protumoral function, inhibition of tumor metastasis, and increased chemosensitivity through regulating the Stat-5/l1r pathway via inhibition of miR-100 in TAMs in a mouse breast cancer model [120]. This further supports the evidence that miRNAs regulate macrophage polarization by altering signaling pathways and transcription factors [121].

The role of miR-146a and miR-222 in TAMs in breast cancer has also been assessed in the similar context [105]. Significant downregulation of miR-146a and miR-222, coordinated with induction of NFκβ-p50 upregulation in TAMs of breast cancer, has been reported. However, conflicting roles of these miRNAs in tumor progression were discovered. Further study revealed that inhibition of miR-146a reduced M2 macrophage markers. As evidence, miR-146a antagomir-transfected macrophages displayed 4T1 tumor mitigation in mice. Their research also observed that overexpression of miR-222 in TAMs targeted CXCL12 and CXCR4 and suppressed macrophage migration and tumor growth in mice [105]. The transcription factor PU.1 has also been shown to target miR-146a, which is involved in differentiation of HSCs to peritoneal macrophages in mice models [114]. Interestingly, estrogen receptor α-expressing breast cancer cells were seen to have increased proliferation upon upregulation of miR-222 within a regulatory loop [122]. Another research group determined M2 macrophages reduced expression of miR-19a-3p and upregulation of the pro-oncogene Fra-1 [123]. Notably, Raw macrophages transfected with a miR-19a-3p mimic increased miR-19a-3p expression in accordance with reduction of the Fra-1 gene and its target genes VEGF, STAT3, and pSTAT. Furthermore, miR-19a-3p mimics decreased 4T1 breast tumor cells migration and invasion by regulating M2 macrophage polarization [123]. Zhong et al. reported downregulation of miR-720 in TAMs of breast cancer [124]. Their research group found that overexpression of miR-720 mitigated the M2 macrophage phenotype and blocked M2 macrophage polarization by targeting GATA-binding protein 3 (GATA3). Increased GATA3 expression has implicated in breast cancer, in situ lesions, and hyperplastic tissue compared to normal breast tissue [125]. Therefore, the role of miRNAs in TAMs is crucial in breast cancer prevention, especially in controlling downstream effects of binding proteins.

Another example would be the higher expression of miRNA-181a in M2 macrophages [126]. Overexpression of miRNA-181a in M1 macrophages inhibited the M1 phenotype and induced the M2 phenotype. In comparison, inhibition of miRNA-181a in M2 macrophages promoted M1 polarization. Importantly, miR-181a inhibition also blocked M2 macrophage-mediated migration and invasion of tumor cells. Mechanistically, miRNA-181a regulates macrophage polarization by targeting Kruppel-like factor 6 (KLF6) and CCAAT/enhancer binding protein-α (C/EBPα) [126]. C/EBPα, a transcription factor that regulates differentiation and cell proliferation, has long been known to be downregulated in primary breast cancers and associated with downregulation of c-myc and upregulation of p21, peroxisome proliferator-activated receptor gamma (PPARγ), and the differentiation marker maspin [127]. Therefore, it is possible that through higher M2 macrophage expression of miRNA-181a, there could be regulatory influences on C/EBPα expression in breast cancer cells.

Moraes et al.’s group assessed the role of immunomodulatory protein Annexin A1 (ANXA1) on macrophage polarization in breast cancer [128]. They found that a lack of Annexin A1 (ANXA1) shifted macrophages toward M1 polarization. Interestingly, lower ANXA1 levels were negatively correlated in ER + MCF-7 breast cancer cells with higher expression levels of oncogenic miR-196a and repression of c-myc and NFκβ [129]. In contrast, ANXA1 absence mitigated tumor 4T1 tumor growth and metastasis in vivo by promoting M1 macrophage polarization [128]. They further revealed that ANXA1 regulates macrophage polarization in the tumor microenvironment by modulating FPR2 (formyl peptide receptor2)-ERK -CCL5 signaling [128].

Recently, inhibitor of differentiation 4 (ID4) was discovered to be expressed at higher levels in triple-negative breast tumors [130]. By increasing the expression of angiogenesis-related genes and suppressing antiangiogenic miR-15b/107, ID4 was also shown to be able to reprogram TAMs [130]. As we know, angiogenesis plays an important role in TAMs and breast cancer expansion, and as such, miRNAs can also introduce negative regulation, with miR-497 inhibiting breast cancer angiogenesis by targeting VEGFR2 [131]. Additional factors secreted from tumors include miR-375, which is secreted by apoptotic breast cancer cells, and were also found in higher levels in TAMs [132]. Frank et al. found that tumor-associated macrophages uptake this miR-375 via CD36 [132]. Furthermore, this miR-375 was shown to be responsible for macrophage migration and infiltration to breast tumor and tumor-promoting microenvironments.

Recent reports have started revealing the significance of miRNA expression in monocytes or macrophages in the TME. It has been demonstrated that miR-146a can control the expansion of inflammatory monocyte precursors and their recruitment to inflamed tissues. Other miRNAs, including miR-20b, -29b, -135a, -150, -155, -342, -424, and -702, are also differentially expressed in monocyte subsets [133], but their relative functions in these cells have yet to be identified. In addition to recruitment, several miRNAs presumably regulate macrophage activation and function in tissues. Most of these miRNAs (miR-155, miR-125a/b, miR-146a, miR-21, and let-7e) in macrophages are upregulated by the Toll-like receptor (TLR) ligands and conserved among mammalian species. Some of these miRNAs can either promote (e.g., miR-155) or suppress (e.g., miR-146a) proinflammatory responses, while some target key regulatory molecules responsible for classical macrophage activation. Recently, it has been suggested that distinct miRNAs, such as miR-187, miR-378-3p, and miR-511-3p, are induced upon alternative macrophage activation.

**Figure 4:**
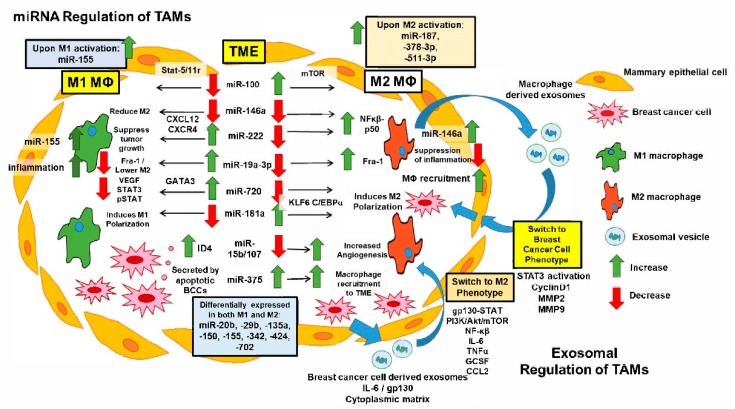
**miRNA and Exosomal Effects in Breast Cancer.** miRNAs manipulate tumor activity inside breast cancer cells through various downstream effects (shown on left). Inflammatory M1 and TAM M2 macrophages (MØ) within the TME show differential effects based on miRNA expression patterns and changes within each MØ phenotype (left-center, black boxes). Green and red arrows indicate up- or down-regulation of various effects, while black arrows indicate pathways. Exosomal production by the BCCs and M2 MØs within the TME has various effects, including M2 polarization, cell invasion, metastatic progression, and protumoral TME progression (right side, blue arrows). Depending on what miRNAs and exosomal effects are present, it would potentially be possible to switch the TAMs phenotype and thus the TME into a more beneficial state for the host.

Studies have shown that interfering with miRNA activity can lead to rewiring the cell activation through targeting important molecular checkpoints that maintain a balance between pro- and anti-tumoral macrophage functions. These findings strengthen the potential and need for the development of pharmacological agents to either suppress or enhance the activity of selected miRNAs with a view to TAMs’ phenotype reprogramming. Several studies reported successful implementation of such approaches by targeting of miR-155 [134], miR-223 [135], and miR-511-3p [136]. Moreover, miRNAs were found to regulate tumor drug resistance. It has been reported that miR-21 reduces the sensitivity of cancer cells to chemotherapeutic drugs while the silencing of this miRNA increases the chemosensitivity by increasing the phosphatase and tensin homolog (PTEN) expression [137,138]. These miRNA effects are summarized in Figure 4.

The above studies clearly indicate that miRNAs play a critical role in breast tumor growth by regulating TAM function. Thus, targeting TAM via miRNA might be an attractive therapeutic means to mitigate breast cancer progression [139,140,141].

## 6. Targeting Exosomal Communication Regulation between Cancer Cells and Macrophages in the TME

Exosomes were initially identified as reticulocytes, 30–100 nm in diameter, and 1.13–1.19 g/mL in density [142]. They originate from multivesicular bodies and are released into the extracellular milieu after fusing multivesicular bodies with the plasma membrane [143]. Exosomes are present in body fluids including blood plasma, saliva, urine, and breast milk. They carry miRNAs, mRNAs, deoxyribonucleic acid (DNA) fragments, and proteins [144]. Evidence suggests that exosomes are released by cancer and normal cells and act as “communication shuttles” between cells that can transduce signals, re-encode genes of target cells, and play an essential role in tumor development, invasion, metastasis, and chemoresistance [145]. As such, exosomes make attractive targets for removal and redirection to halt tumor formation. Our current knowledge of how exosomes impact breast tumor growth and metastasis by influencing the TAM function has been summarized in Figure 4.

Increasing evidence suggests that exosomes influence tumor progression by modulating the TME [146,147,148,149,150,151,152]. Higher levels of interleukin-6 (IL-6) receptor beta (glycoprotein 130, gp130) in breast cancer cell exosomes were reported by Ham et al. [153]. Bone-marrow-derived macrophages (BMDMs) exposed to breast cancer cell-derived exosomes demonstrated a phenotype change in BMDMs, similar to TAMs, which was mediated via the gp130-STAT3 pathway. Their group further established that gp130 inhibition mitigated the effects of exosomes on BMDMs [153].

Another research group reported that circulating exosomes secreted by breast cancer cells increased macrophage NF-κB activation and induced proinflammatory activity by upregulation of inflammatory cytokines IL-6, TNFα, GCSF, and CCL2 [154]. This increase in inflammatory responses provided a tumor-promoting environment by regulating Toll-like receptor 2 (TLR2) [154]. These studies indicate that cancer-cell-derived exosomes play a critical role in tumor progression by activating and altering the functions of macrophages.

The role of macrophage-derived exosomes was assessed in the initiation and progression of metastasis following chemotherapy in a study by Yu et al. Their group established a postchemotherapy cancer microenvironment model using THP-1-derived macrophages cocultured with apoptotic breast cancer cells (MCF-7 or MDA-MB-231) to mimic an in vivo microenvironment [155]. They found that exosomes isolated from macrophages after coculture with apoptotic breast cancer cells demonstrated the ability to induce proliferation, invasiveness, and metastasis of breast cancer in vitro and in vivo by activating STAT3 and its target genes CyclinD1, MMP2, and MMP9. This could be related to the earlier study by Yang et al., where decreased levels of miRNA-19a-3p and increased levels of proto-oncogene Fra-1 were detected in M2-polarized macrophages [123]. Increased levels of Fra-1 have been implicated with increased expression of its target genes VEGF, STAT3, and pSTAT [123].

Li et al. investigated the relationship between TAM and endocrine-resistant phenotype of breast cancer. They exposed macrophages with cytoplasmic matrix (CM) from tamoxifen-sensitive (MCF7-S) or -resistant (MCF7-R) MCF7 breast cancer cells [156]. The CM from tamoxifen-sensitive (MCF7-S) breast cancer cells showed a higher potency to induce M2 type phenotype. This phenotypic change of macrophages was associated with higher expression of chemokine ligand 2 and activation of PI3K/Akt/mTOR pathway. This is noteworthy, as stated above, because overexpression of miR-100 promoted the TAMs phenotype by regulating the mTOR pathway [120]. Perhaps, increased levels of miR-100 are present in the cytoplasmic matrix introduced from the TAMs.

Exosomes also can modulate the TME by delivering miRNA. TAMs facilitate exosome-mediated delivery of invasion-potentiating miRNAs to breast cancer to promote the invasion and metastasis of breast cancer [135]. Exosomes containing miRNAs also play a critical role to regulate TME and tumor progression. Jang et al. found that epigallocatechin gallate (EGCG) can upregulate miR-16 in breast tumor cells and, upon transferring this miRNA to TAMs via exosomes, inhibits TAM infiltration and M2 polarization by reducing expression of IL-6 and TGF-β and increasing TNF-α expression [157]. Human plasma containing breast cancer exosomes contained higher levels of miR-1246 and miR-21 compared to the plasma exosomes of healthy control subjects, in a study by Hannafon et al. [158]. Exosomal miR-128 has been shown to reduce the expression of Bcl-2-associated X protein (Bax) in treated breast cancer cells, in a study by Wei et al. [159]. In a similar study by Eichelser et al., miR-373 was shown to repress the expression of estrogen receptor and inhibit apoptosis of breast cancer cells as well [160]. Exosomes not only modulate the phenotype of tumor cells but also other stromal cells present in the TME. However, future studies are needed to minimize the negative effects of tumor-derived exosomes on tumors and how to use exosomes to deliver anticancer drugs effectively in clinical practice.

## 7. Conclusions and Future Perspectives

Increasing TAMs are intimately associated with tumor progression by inducing angiogenesis, facilitating metastasis, and suppressing the immune system. In solid tumors, the phenotypic characterization of TAMs revealed the presence of M2-type macrophages and the absence of M1 phenotype. Therefore, promoting TAM repolarization toward the M1 phenotype without causing significant side effects and depletion of tumor-supporting macrophages may show therapeutic potential against solid tumors. Pharmacological or chemical agents targeting TAMs have recently developed, and clinical trials using some of these agents are still in progress. Moreover, studies showed that miRNAs influence breast tumor growth by modulating both TAM recruitment and polarization. More studies are required to investigate the efficacy of two or more miRNA combinations on macrophage polarization and in the regulation of TAMs function, which may provide decisive clues in developing new promising therapies against breast cancer. Exosomes also play a critical role in regulating angiogenesis and tumor metastasis. However, knowledge about the effects of exosomes secreted by cancer cells vs. TAMs and their impacts on tumor progression remains unknown. Future studies are required to optimize the exosome isolation procedure and its use as a drug delivery system to treat cancer in a clinical setting. Overall, altering TAM function in the tumor microenvironment is an attractive approach to treat breast tumors. However, a leading issue that needs to be resolved is how to deliver TAM targeted therapy that modulates the functions of tumor-promoting macrophages without blocking the antitumor activity of macrophages. Thus, more studies are required to develop effective TAM-targeted therapies.

## Abbreviations

AKT	Protein kinase B
bFGF	Basic fibroblast growth factor
BMDM	Bone marrow derived macrophages
CCL2	C-C motif chemokine ligand 2
CCR2	C-C motif chemokine receptor 2
CD	Cluster of differentiation
CSCs	Cancer stem cells
CSF	Colony-stimulating factor
CSF1R	Colony stimulating factor 1 receptor
CTLA4	Cytotoxic T-lymphocyte-associated protein 4
CXCL12	CXC motif chemokine ligand 12
CXCR4	CXC chemokine receptor 4
C/EBP	CCAAT-enhancer-binding protein
DNA	Deoxyribonucleic acid
ECM	Extracellular matrix
EGCG	Epigallocatechin gallate
EGF	Epidermal growth factor
EGFR	Epidermal growth factor receptor
ER	Estrogen receptor
HB-EGF	Heparin- binding EGF-like growth factor
HER2	Human epidermal growth factor receptor 2
HIFs	Hypoxia-inducible-factors
HUVEC	Human umbilical vein endothelial cells
HSP	Heat shock proteins
ICAM-1	Intracellular adhesion molecule-1
IFN-ƴ	Interferon gamma
ILs	Interleukins
IL4Rα	IL-4 receptor α
iNOS	Inducible nitric oxide synthase
IRF	Interferon regulatory factor
KLF	Kruppel-like factor
LPS	Lipopolysaccharide
mAbs	Monoclonal antibodies
M-CSF	Macrophage colony-stimulating factor
MDSCs	Myeloid-derived suppressor cells
MHC	Major histocompatibility complex
MIF	Macrophage inhibitory factor
miRNA	MicroRNA
MMP9	Matrix metalloproteinase 9
MYC	MYC Proto-Oncogene, BHLH Transcription Factor
NF-kβ	Nuclear factor—kappa beta
NK	Natural killer
NO	Nitric oxide
PDGF	Platelet-derived growth factor
PI3K	Phosphatidylinositol-3-kinase
PPARƴ	Peroxisome proliferator-activated receptor gamma
PTEN	Phosphatase and tensin homolog
RNA	Ribonucleic acid
shRNA	Small hairpin RNA
siRNA	Short-interfering RNA
SOX	SRY-related HMG-box
STAT3	Signal transducer and activator of transcription 3
TAMs	Tumor-associated macrophages
TDEs	Tumor-derived exosomes
TGF-β	Transforming growth factor beta
TIE2	TEK tyrosine kinase endothelial
TLR	Toll-like receptor
TME	Tumor microenvironment
TNFα	Tumor necrosis factor alpha
TRAIL	TNF-related apoptosis-inducing ligand
Tregs	Regulatory T cells
VEGF	Vascular endothelial growth factor
VEGFR	Vascular endothelial growth factor receptor
